# Supplementary material for: Making thermodynamic models of mixtures predictive by machine learning: matrix completion of pair interactions
Source: Chem Sci. 2022 Apr 4;13(17):4854–62. doi: 10.1039/d1sc07210b (PMC9067573; doi:10.1039/d1sc07210b)
Supplement: SC-013-D1SC07210B-s002 [file SC-013-D1SC07210B-s002.pdf]

Electronic Supplementary Material (ESI) for Chemical Science.
This journal is © The Royal Society of Chemistry 2022

Supporting Information for: Making Thermodynamic Models of Mixtures Predictive by Machine-Learning of Pair Interactions

Fabian Jirasek,[*,†] Robert Bamler,[‡] Sophie Fellenz,[¶] Michael Bortz,[§] Marius Kloft,[¶] Stephan Mandt,[‖,⊥] and Hans Hasse[†,⊥]

†*Laboratory of Engineering Thermodynamics (LTD), TU Kaiserslautern, Erwin-Schrödinger-Str. 44, 67663 Kaiserslautern, Germany*

‡*Data Science and Machine Learning, University of Tübingen, Maria-von-Linden-Str. 6, 72076 Tübingen, Germany*

¶*Machine Learning Group, TU Kaiserslautern, Paul-Ehrlich-Str. 34, 67663 Kaiserslautern*

§*Fraunhofer Institute for Industrial Mathematics (ITWM), Fraunhofer-Platz 1, 67663 Kaiserslautern, Germany*

‖*Department of Computer Science, University of California, 92617 Irvine, CA, USA*

⊥*Stephan Mandt and Hans Hasse jointly supervised this work.*

E-mail: fabian.jirasek@mv.uni-kl.de

UNIQUAC Equations

UNIQUAC models the logarithmic activity coefficient $\ln \gamma_i$ of a component i in a mixture as a combination of a combinatorial part (C) and a residual part (R):[1,2]

$$\ln \gamma_i = \ln \gamma_i^{\mathrm{C}} + \ln \gamma_i^{\mathrm{R}} \tag{S.1}$$

The combinatorial part $\ln \gamma_i^{\mathrm{C}}$ is calculated by:

$$\ln \gamma_i^{\mathrm{C}} = 1 - V_i + \ln V_i - \frac{z}{2} q_i \left(1 - \frac{V_i}{F_i} + \ln \frac{V_i}{F_i}\right) \tag{S.2}$$

with

$$V_i = \frac{r_i}{\sum_j r_j x_j} \tag{S.3}$$

$$F_i = \frac{q_i}{\sum_j q_j x_j} \tag{S.4}$$

where r_i and q_i are the relative Van der Waals volume and surface area of component i, respectively, x_i is the mole fraction of i in the mixture, and z is the coordination number, which is usually set to 10; $z = 10$ was also used throughout this work. Hence, $\ln \gamma_i^{\mathrm{C}}$ depends, besides on the composition, only on pure-component parameters of i and the ones of the remaining components $j = 1..J$ that make up the mixture that is considered.

The residual part $\ln \gamma_i^{\mathrm{R}}$ is calculated by:

$$\ln \gamma_i^{\mathrm{R}} = q_i \left(1 - \ln \frac{\sum_j q_j x_j \tau_{ji}}{\sum_j q_j x_j} - \sum_j \frac{q_j x_j \tau_{ij}}{\sum_k q_k x_k \tau_{kj}}\right) \tag{S.5}$$

with

$$\tau_{ij} = \exp\left(-\frac{\Delta U_{ij}}{RT}\right); \qquad \tau_{ji} = \exp\left(-\frac{\Delta U_{ji}}{RT}\right) \tag{S.6}$$

where ΔU_{ij} and ΔU_{ji} are pair-interaction parameters describing the pairwise interaction between the two components i–j in the mixture, R is the universal gas constant, and T is the thermodynamic temperature in Kelvin. Hence, in contrast to the combinatorial part $\ln \gamma_i^{\mathrm{C}}$, the residual part $\ln \gamma_i^{\mathrm{R}}$ depends on both pure-component parameters (q_i) and pair-interaction parameters between all combinations of components in the mixture (ΔU_{ij}, ΔU_{ji}).

The pure-component parameters (r_i, q_i) are reported for a large number of relevant components, and can also be estimated with the approach introduced in combination with the

group-contribution method UNIFAC.[3,4] The pair-interaction parameters (ΔU_{ij}, ΔU_{ji}), on the other hand, which are required for all binary combinations i–j of the components that make up the considered mixture, are usually fitted to experimental data on the respective binary subsystem i–j, oftentimes to data on the vapor-liquid equilibrium (VLE). Since experimental mixture data are, however, in general scarce, ΔU_{ij} and ΔU_{ji} have so far only been reported for a small fraction of the practically relevant systems.

In basically all cases when UNIQUAC is used in practice, ΔU_{ij} and ΔU_{ji} are *individually* fitted to data for the respective binary system i–j. Following the derivation of UNIUQAC[1,2] from the lattice theory, these parameters are, however, not independent across binary systems, but are linked via pair-interaction energies U:

$$\Delta U_{ij} = U_{ij} - U_{jj} \tag{S.7a}$$

$$\Delta U_{ji} = U_{ij} - U_{ii} \tag{S.7b}$$

where U_{ii} and U_{jj} are like pair-interaction energies of the components i and j, respectively, i.e., they describe the pairwise interaction of molecules of the same type (i–i or j–j), whereas U_{ij} is an unlike pair-interaction energy that describes the pairwise interaction of different molecules (i–j here). U_{ij} is, in contrast to ΔU_{ij}, by definition symmetric, i.e., in general it holds that

$$U_{ij} = U_{ji} \tag{S.8}$$

but

$$\Delta U_{ij} \neq \Delta U_{ji}. \tag{S.9}$$

By fitting ΔU_{ij} and ΔU_{ji} for multiple systems *individually* to data for each binary systems i–j, the resulting parameters will almost certainly not comply with the physical constraint given in Eq. (S.7). As an example, let us consider three different components, which we simply number consecutively (1, 2, 3), and all binary combinations of them (1–2, 1–3, 2–

3). According to Eq. (S.7) and using Eq. (S.8), the resulting six pair-interaction parameters of UNIQUAC are correlated via:

$$\Delta U_{12} = U_{12} - U_{22} \tag{S.10a}$$

$$\Delta U_{21} = U_{12} - U_{11} \tag{S.10b}$$

$$\Delta U_{13} = U_{13} - U_{33} \tag{S.10c}$$

$$\Delta U_{31} = U_{13} - U_{11} \tag{S.10d}$$

$$\Delta U_{23} = U_{23} - U_{33} \tag{S.10e}$$

$$\Delta U_{23} = U_{23} - U_{22} \tag{S.10f}$$

Pairwise aggregating yields:

$$\Delta U_{12} + U_{22} = \Delta U_{21} + U_{11} \tag{S.11a}$$

$$\Delta U_{13} + U_{33} = \Delta U_{31} + U_{11} \tag{S.11b}$$

$$\Delta U_{23} + U_{33} = \Delta U_{32} + U_{22} \tag{S.11c}$$

By subtracting Eq. (S.11c) from Eq. (S.11b), we obtain:

$$\Delta U_{13} - \Delta U_{23} = \Delta U_{31} - \Delta U_{32} + U_{11} - U_{22} \tag{S.12}$$

Finally, substituting U_{11} by Eq. (S.11a) in Eq. (S.12) and simplifying yields:

$$\Delta U_{13} - \Delta U_{23} = \Delta U_{31} - \Delta U_{32} + \Delta U_{12} - \Delta U_{21} \tag{S.13}$$

Eq. (S.13) will almost certainly not be satisfied if the pair-interaction parameters are fitted to the respective binary systems *individually*, as it is usually done in practice. In other words, UNIQUAC is commonly applied in a way in which the complete set of pair-interaction parameters for N components can be arranged in an asymmetric $N \times N$ matrix

with empty diagonal. However, following the derivation of UNIQUAC, only a triangular matrix (or a symmetric matrix) can be filled with truly independent parameters, namely the pair-interaction energies U, with the like energies U_{ii} representing the diagonal and the unlike energies U_{ij} the off-diagonal elements.

Data and Data Preprocessing

Data set

All data points used in this work were taken from the Dortmund Data Bank (DDB) 2020.[5] For training the model, only data for binary mixtures were used. For evaluating the performance, also mainly data for binary mixtures were used, but, in addition, also some data sets for ternary mixtures were considered as described below. In general, only data for mixtures of molecular components were used, while data for mixtures containing salts, ionic liquids, pure metals, or unspecified components were excluded. Also excluded were all data sets that were labeled as questionable or of poor quality in the DDB. Further, only components i were considered for which the relative van der Waals volume and surface area, r_i and q_i, respectively, were either reported in the DDB or could be calculated with the approach described in connection with original UNIFAC[3] in its present parameteriziation.

Two types of data for binary mixtures reported in the DDB were used: first, data on activity coefficients at infinite dilution in binary mixtures γ_{ij}^{∞} and second, data on the vapor-liquid equilibrium (VLE) of binary mixtures up to a pressure of 10 bar. From the VLE data, activity coefficients γ_{ij} (at finite concentration) were calculated using extended Raoult's law assuming an ideal gas phase and neglecting the pressure dependence of the chemical potential in the liquid phase:

$$p_i^{\mathrm{s}}(T) \cdot x_i \cdot \gamma_{ij}(T, x_i) = p \cdot y_i \tag{S.14}$$

where p_i^{s} is the vapor pressure of the pure component i at the considered temperature T, x_i

and y_i are the mole fraction of i in the liquid phase and in the vapor phase, respectively, γ_{ij} is the activity coefficient of i in the liquid mixture (with j), and p is the pressure. p_i^{s} was calculated with the Antoine equation using Antoine parameters reported in the DDB, if available. A VLE data point from the DDB was therefore only used if information on T, x_i, y_i, and p were reported and if Antoine parameters for the considered component i and in the relevant temperature range were available in the DDB. Also VLE data for mixtures with formic acid and acetic acid were excluded from the data set as these components can usually not be modeled with an ideal vapor phase even at low to moderate pressures. Eq. S.14 and the named pure-component parameters were also used for the prediction of VLE phase diagrams from the predicted γ_{ij} after training the model.

The γ_{ij} calculated from the VLE data according to Eq. S.14 were, together with the data for γ_{ij}^{∞} adopted from the DDB, further processed as described in the following. If multiple data points for the same system i–j, the same concentration x_i, *and* same temperature T were available, the median of the activity coefficients was calculated and used. Further, only the data for those systems i–j were used for which at least two distinct data points, i.e., at different temperatures and/or concentrations, were available. The latter constraint is not necessary for the hybrid model MCM-UNIQUAC introduced here, but it is required for the classical system-wise fitting of UNIQUAC, which was used as benchmark here, cf. Figure 2 in the manuscript.

Finally, each component for which data in *only a single* binary system were available, was rejected, resulting in a data set in which:

- for each component i, j data for at least two different binary systems i–j, and
- for these systems, at least two data points (at different temperature and/or concentration) were available.

The resulting data set comprises 363181 experimental data points for 12199 binary systems i–j including 1146 components i, j and covers a temperature range from 183 K to 638 K.

This data set was split into a training set containing all data points for 80% (9759) of the binary systems i–j, a validation set containing all data points for 10% (1220) of the binary systems, and a test set containing all data points for the remaining 10% (1220) of the binary systems. This data split was done randomly, but ensuring that for each component i, j, data on at least one binary system i–j were available in the training set. This is necessary as the training set was subsequently used for learning the parameters of MCM-UNIQUAC, which requires (at least some) information for each component. The validation set was used for model selection, i.e., for choosing the model's hyperparameters (see below). Therefore, the model was trained on the training set for different hyperparameters and the predictions for the data from the validation set were compared to the (true) experimental data. Based on the resulting mean absolute error (MAE) and mean squared error (MSE), the best hyperparameters were chosen and the "final" model was evaluated based on the MAE and MSE scores on the test set, which was used neither for training the model nor for choosing the hyperparameters. In all cases, the MAE and MSE scores were calculated by averaging over binary systems; specifically, we calculated the MAE (MSE) for each binary system from the respective set by comparing the logarithmic activity coefficients $\ln \gamma_{ij}$ as predicted with MCM-UNIQUAC to the respective experimental values from the DDB, and subsequently averaging the obtained MAE (MSE) over all systems. The same procedure was applied with the predictions of UNIFAC for obtaining the respective scores for comparison.

Model Details

Bayesian Matrix Completion

As in our previous works,[6,7] we use a Bayesian approach to matrix completion for the prediction of logarithmic activity coefficients $\ln \gamma_{ij}$ in unmeasured binary mixtures i–j in the present work. The approach consists of three steps. In the first step, a generative probabilistic model for the variable of interest, i.e., $\ln \gamma_{ij}$ here (the logarithm was used for scaling

purposes), is specified as a nonlinear function of the components i, j, the temperature T, and the composition of the mixture given by the mole fraction of component i x_i, as well as relative van der Waals volumes q_i and q_j and surface areas r_i and r_j of the components i and j (taken from the DDB[5] if available or calculated with the approach introduced in combination with original UNIFAC[3,4] otherwise, cf. above), initially unknown (latent) features vectors θ_i, θ_j, β_i, and β_j of the components, and the like pair-interaction energies U_{ii} and U_{jj} of the components. The nonlinear function is defined by the UNIQUAC model, cf. Eqs. (S.1)-(S.6), but additionally acknowledges the correlations of the pair-interaction parameters ΔU via pair-interaction energies U as follows from the derivation of UNIQUAC, cf. Eq. (S.7). The vectors θ_i, θ_j, β_i, and β_j, which contain the latent features of the components and model the unlike pair-interaction energies, cf. Eq. (4) in the manuscript, and whose length K is a hyperparameter that was set to $K = 3$ during model selection, as well as the like pair-interaction energies U_{ii} and U_{jj} constitute the parameters of our model and are inferred during the training step.

For inferring the model parameters, the probabilistic model defines a probability distribution over all $\ln \gamma_{ij}$ from the training set by specifying a stochastic process that generates hypothethical data on $\ln \gamma_{ij}$ conditioned on the initially unknown (latent) parameters of the components $(\theta_i, \theta_j, \beta_i, \beta_j, U_{ii}, U_{jj})$, known descriptors of the pure components (q_i, q_j, r_i, r_j), and specified conditions (T, x_i), which are subsequently "compared" to the true experimental data (at the same conditions). Therefore, the generative process first draws latent parameters from a normal prior distribution with zero mean and a standard deviation of one; mean and standard deviation of this prior distribution are also hyperparameters of the model and were set based on the scores on the validation set. The generative process then models the probability of the experimental activity coefficients $\ln \gamma_{ij}^{\text{exp}}$ from the training set as a Cauchy likelihood distribution with scale λ centered around the results of the UNIQUAC equations with the latent parameters drawn from the prior, the given known descriptors of the components, and the specified conditions; the scale λ, which was set to $\lambda = 0.2$, as well as the

choice of a Cauchy distribution are also hyperparameters of the model and were selected based on the scores on the validation set. The likelihood can be written as follows:

$$\ln \gamma_{ij}^{\text{exp}}(T, x_i) = \text{Cauchy}\left(f_{\text{UNIQUAC}}\left(U_{ij}, U_{ii}, U_{jj}, q_i, q_j, r_i, r_j, T, x_i\right), \lambda\right) + \epsilon_{ij} \tag{S.15}$$

where f represents Eqs. S.1-S.7, U_{ij} is calculated according to Eq. 4 in the manuscript, and the random variable ϵ_{ij} captures both inaccuracies of the experimental data and the model.

In the second step, the latent parameters (θ_i, θ_j, β_i, β_j, U_{ii}, U_{jj}) are simultaneously inferred for all considered components during the training of the specified generative model on all experimental data for $\ln \gamma_{ij}$ from the training set by inverting the generative model. For this purpose, we resorted to Gaussian mean-field Variational Inference[8–10] as this has shown good results in our previous works.[6,7] Since the generative model is probabilistic, all inferred latent parameters are random variables, and for each latent parameter, a probability distribution, called posterior, is obtained.

In the third step, we use the means of the approximate posterior distributions over θ_i, θ_j, β_i, and β_j to predict the unlike pair-interaction energies U_{ij} according to Eq. 4 in the manuscript, which we then use together with the means of the approximate posterior distributions over U_{ii} and U_{jj} to predict the pair-interaction parameters ΔU_{ij} and ΔU_{ji} of UNIQUAC according to Eq. S.7, which we, finally, use to predict temperature- and concentration-dependent $\ln \gamma_{ij}$ with Eqs. S.1-S.6 by also using the known geometric pure-component parameters (q_i, q_j, r_i, r_j).

For all data points in the test set, the predictions for $\ln \gamma_{ij}$ are compared to the respective experimental values to evaluate the predictive performance of the model. For performing the above described steps, we use the Stan framework[11] that allows specifying user-defined generative models and automates the task of Bayesian inference.[10] Figure S.1 shows the code for the probabilistic model in Stan.

```
data {
    int<lower=0> I;                         // number of components
    int<lower=0> N;                         // number of data points in training set
    int<lower=0> K;                         // number of latent dimensions
    real<lower=0> Factor;                   // factor for scaling the latent parameters (set to 1000 here)
    real Data[N,3];                         // list of data points: temperature T, mole fraction of i x_i, ln_gamma_ij
    int<lower=0> Components[N, 2];          // list of component identifiers: i, j
    real<lower=0> Parameters[I, 2];         // list of geometric pure-component parameters: r, q
    real mu_0;                              // prior mean
    real<lower=0> sigma_0;                  // prior standard deviation
    real<lower=0> lambda;                   // likelihood scale
}

parameters {
    vector[K] theta[I];         // feature vectors for unlike interaction energies
    vector[K] beta[I];          // feature vectors for unlike interaction energies
    real pure[I];               // pure-component energies
}

model {
    int i;                      // solute identifier
    int j;                      // solvent identifier
    real T;                     // temperature in K
    real conc1;                 // mole fraction of i
    real conc2;                 // mole fraction of j
    real r1;                    // r_i
    real r2;                    // r_j
    real q1;                    // q_i
    real q2;                    // q_j
    real V1;                    // volume fraction of i per mixture mole fraction
    real F1;                    // surface area fraction of i per mixture mola fraction
    real u11;                   // binary pure-component interaction energy i-i
    real u22;                   // binary pure-component interaction energy j-j
    real u12;                   // binary mixture interaction energy i-j (symmetric)
    real Delta_u12;             // UNIQUAC interaction parameter i-j
    real Delta_u21;             // UNIQUAC interaction parameter j-i
    real tau12;                 // temperature-dependent interaction parameter i-j
    real tau21;                 // temperature-dependent interaction parameter j-i

    // Prior: draw latent parameters for all components:
    for (k in 1:I) {
        theta[k] ~ normal(mu_0, sigma_0);
        beta[k] ~ normal(mu_0, sigma_0);
        pure[k] ~ normal(mu_0, sigma_0);
    }

    // Likelihood: model the propability of the experimental ln_gamma_ij as a Cauchy distribution
    // around the UNIQUAC result based on the interaction parameters calculated from the latent parameters
    for (r in 1:N) {
        i = Components[r, 1];
        j = Components[r, 2];
        T = Data[r, 1];
        conc1 = Data[r, 2];
        conc2 = 1-conc1;
        r1 = Parameters[i, 1];
        q1 = Parameters[i, 2];
        r2 = Parameters[j, 1];
        q2 = Parameters[j, 2];
        u12 = (theta[i]' * beta[j]) + (theta[j]' * beta[i]);
        u11 = pure[i];
        u22 = pure[j];
        Delta_u12 = (u12 - u22) * Factor;
        Delta_u21 = (u12 - u11) * Factor;
        V1 = r1 / (conc1 * r1 + conc2 * r2);
        F1 = q1 / (conc1 * q1 + conc2 * q2);
        tau12 = exp(-Delta_u12 / (T * 8.314));          // universal gas constant R = 8.314 J/(mol*K)
        tau21 = exp(-Delta_u21 / (T * 8.314));
        Data[r, 3] ~ cauchy(
            1 - V1 + log(V1) - 5*q1*(1-V1/F1+log(V1/F1)) + q1*(1-log((q1*conc1+q2*conc2*tau21)/(q1*conc1+q2*conc2))...
            - (q2*conc2*tau12)/(q2*conc2+q1*conc1*tau12) - (q1*conc1)/(q1*conc1+q2*conc2*tau21)),
            lambda
        );
    }
}
```

Figure S.1: Stan code of the probabilistic generative model for the prediction of activity coefficients by embedding an MCM into the thermodynamic model UNIQUAC.

Additional Results

In Figure S.2 (left), the results of MCM-UNIQUAC to describe the $\ln \gamma_{ij}$ from the training set and to predict the $\ln \gamma_{ij}$ from the test set are shown by considering the mean squared error (MSE) averaged over binary systems. In Figure S.2 (right), the results of MCM-UNIQUAC are compared to those of the physical baseline method modified UNIFAC (Dortmund)[12,13] (UNIFAC), whereby only those data points from the training set or test set were considered that could also be modeled by UNIFAC ('UNIFAC horizon'). The respective results for the mean absolute error (MAE) are shown in Figure 2 in the manuscript; MCM-UNIQUAC clearly outperformes UNIFAC in both metrics.

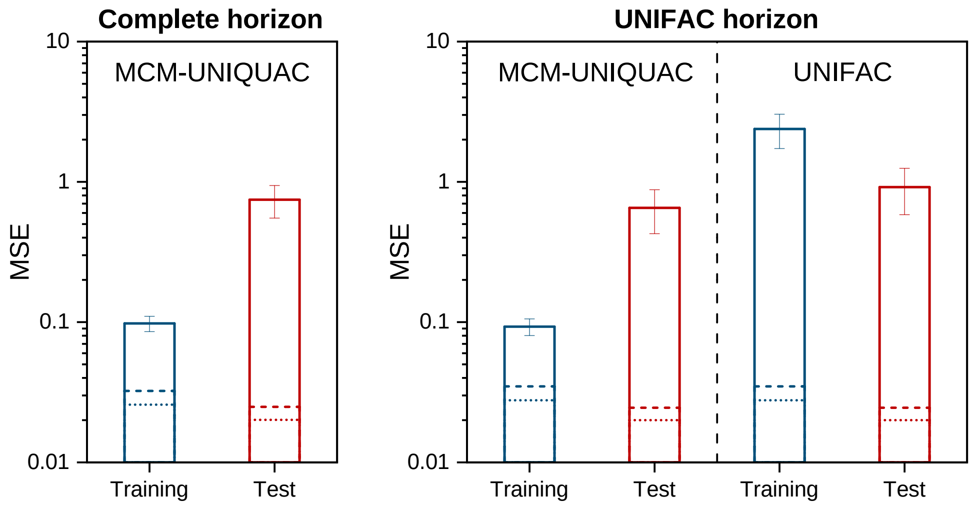

Figure S.2: Mean squared error (MSE) of MCM-UNIQUAC on the training and test set (left) and comparison to UNIFAC based only on those systems that can also be modeled with UNIFAC (right). The bars indicate the results of MCM-UNIQUAC and UNIFAC, whereas the lines denote the baselines obtained by directly fitting UNIQUAC pair-interaction parameters (ΔU_{ij}, dotted) or pair-interaction energies (U_{ij}, dashed) to all available data points. Error bars denote standard errors of the means.

In Figure S.3, the predictions of MCM-UNIQUAC and UNIFAC for the data from the test set are compared in a histogram representation. For a fair comparison of the two methods, only those data points that can be predicted with both methods are considered here. Figure S.3 shows the mean absolute error (MAE, left) and the mean squared error (MSE, right) of MCM-UNIQUAC and UNIFAC for each binary system as calculated by comparing the

predictions for $\ln \gamma_{ij}$ to the respective experimental data; as in Figure 2 in the manuscript, all scores were obtained by averaging over binary systems.

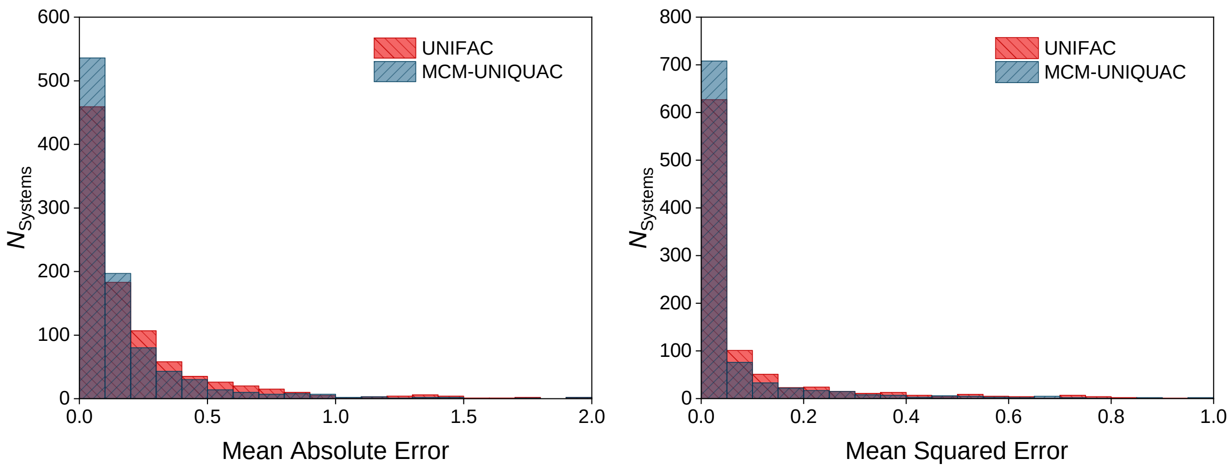

Figure S.3: Mean absolute error (MAE, left) and mean squared error (MSE, right) of the predictions for $\ln \gamma_{ij}$ for binary systems from the test set with MCM-UNIQUAC and UNIFAC in histogram representations. The deviations were calculated by comparing the predictions to the respective experimental values from the DDB. The shown ranges for the MAE (left) cover 98.3% of all systems for MCM-UNIQUAC and 98.0% for UNIFAC. The shown ranges for the MSE (right) cover 95.4% of all systems for MCM-UNIQUAC and 94.7% for UNIFAC.

The results show a better performance of MCM-UNIQUAC compared to UNIFAC, i.e., there are more binary systems for which $\ln \gamma_{ij}$ can be predicted accurately (with low MAE and MSE) than this is the case with UNIFAC.

For the sake of completeness, Figure S.4 shows histogram representations similar to those shown in Figure S.3, but only considering the predictions with MCM-UNIQUAC for those data points from the test set that can *not* be modeled with UNIFAC.

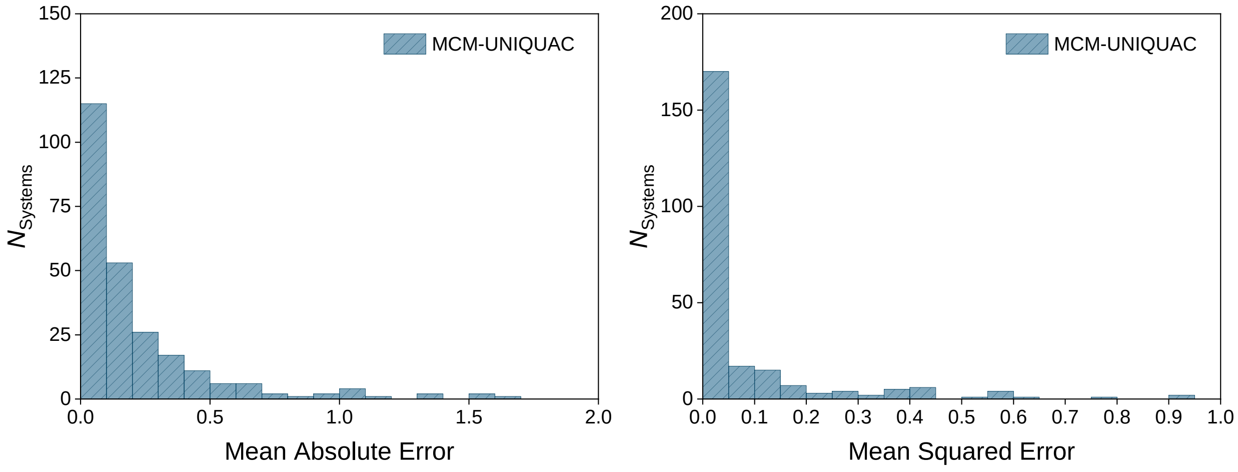

Figure S.4: Mean absolute error (MAE, left) and mean squared error (MSE, right) of the predictions of MCM-UNIQUAC for $\ln \gamma_{ij}$ in the binary systems from the test set that can *not* be modeled with UNIFAC in histogram representations. The deviations were calculated by comparing the predictions to the experimental data from the DDB. The shown ranges cover 96.1% (MAE, left) and 91.9% (MSE, right) of all systems.

The performance of MCM-UNIQUAC for the prediction of $\ln \gamma_{ij}$ for those systems is slightly worse than that for the systems that can also be modeled with UNIFAC, cf. Figure S.3. However, again for most systems small MAE and MSE scores are observed, indicating a reasonable accuracy of MCM-UNIQUAC. We attribute the slightly worse accuracy of MCM-UNIQUAC for the systems considered here to the fact that these mostly relate to rather uncommon components, which are less studied and, as a consequence, for which less data points for training the model are available.

Comparison of MCM-UNIQUAC to COSMO-SAC-dsp

In Figure S.5, we additionally compare the predictions of MCM-UNIQUAC to those of another baseline method, namely the quantum-chemical based COSMO-SAC-dsp.[14,15] Similar to Figure S.3, only the predictions for those data from the test set that can also be predicted with COSMO-SAC-dsp are considered in Figure S.5 for a fair comparison; the σ-profiles for the COSMO-SAC-dsp predictions were taken from Ref.[15]

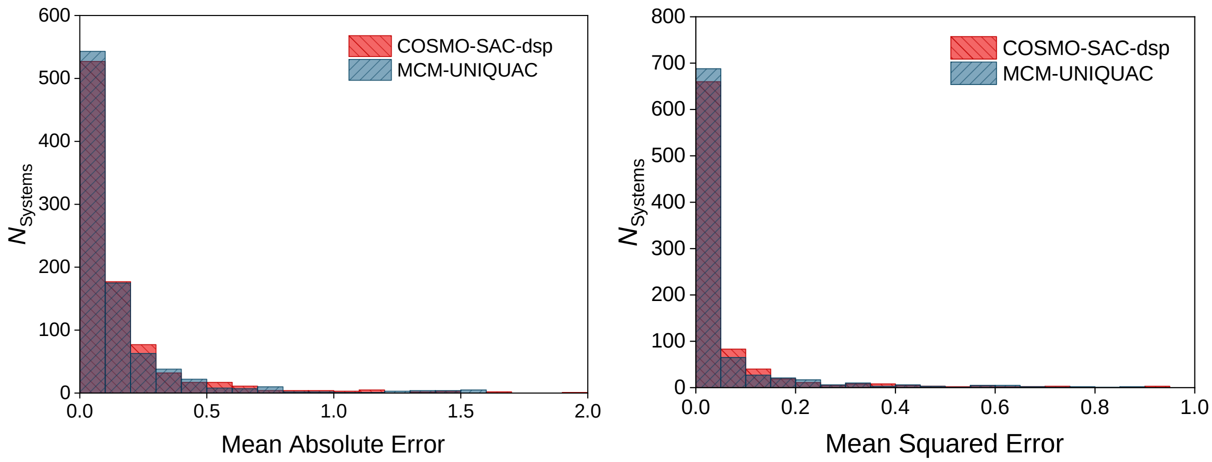

Figure S.5: Mean absolute error (MAE, left) and mean squared error (MSE, right) of the predictions for $\ln \gamma_{ij}$ for binary systems from the test set with MCM-UNIQUAC and COSMO-SAC-dsp[14,15] in histogram representations. The deviations were calculated by comparing the predictions to the respective experimental values from the DDB. The shown ranges for the MAE (left) cover 99.3% of all systems for MCM-UNIQUAC and 99.1% for COSMO-SAC-dsp. The shown ranges for the MSE (right) cover 96.8% of all systems for MCM-UNIQUAC and 96.2% for COSMO-SAC-dsp.

The left panel of Figure S.5 shows the MAE of MCM-UNIQUAC and COSMO-SAC-dsp for each binary system as calculated by comparing the predictions for $\ln \gamma_{ij}$ to the respective experimental data from the DDB, whereas the right panel shows the respective results for the MSE of both methods. Again, we find a better performance of MCM-UNIQUAC, which predicts more systems with smaller MAE and MSE. The average MAE is 0.177 for MCM-UNIQUAC and 0.193 for COSMO-SAC-dsp, the average MSE is 0.354 for MCM-UNIQUAC and 0.491 for COSMO-SAC-dsp. For comparison, the average MAE over all systems from the test set that can be predicted with UNIFAC, cf. Figure S.3, is 0.222 for MCM-UNIQUAC and 0.298 for UNIFAC; the respective MSE scores are 0.380 for MCM-UNIQUAC and 0.917 for UNIFAC.

While the scores of MCM-UNIQUAC are better than that of both baselines throughout, COSMO-SAC-dsp shows better results for the MAE and MSE compared to UNIFAC here. However, note that the scopes of UNIFAC and COSMO-SAC-dsp are different: with the current version of UNIFAC 78.8% of the systems form the test set can be predicted, while

this is the case for only 73.3% of the systems with COSMO-SAC-dsp based on the σ-profiles reported in Ref.[15] And there are in general systems from the test set that can only be modeled by either UNIFAC or COSMO-SAC-dsp; the fact that also the scores of MCM-UNIQUAC are lower for the 'COSMO-SAC-dsp set', cf. Figure S.5, than for the 'UNIFAC set', cf. Figure S.3, indicates that UNIFAC covers at least some systems that are more difficult to describe. However, we consider a more detailed discussion of the advantages of UNIFAC over COSMO-SAC-dsp and vice versa out of the scope of the present work.

Influence of the Number of Training Data

In Figure S.6, we evaluate the influence of the number of training data points for each component on the predictive accuracy of MCM-UNIQUAC. Therefore, the MAE (left) and the MSE (right) of the MCM-UNIQAC predictions for the binary systems $i - j$ from the test set are plotted as a function of the minimum number of systems in the *training set*, in which the components i and j are present, which is called $\min(N_i^{(\mathrm{s})}, N_j^{(\mathrm{s})})$ here. Hence, for small $\min(N_i^{(\mathrm{s})}, N_j^{(\mathrm{s})})$, there are, at least for one of the components i and j, only few systems in the training set containing this component; on the other hand, if $\min(N_i^{(\mathrm{s})}, N_j^{(\mathrm{s})})$ is large, both i and j are well represented in the training set. We can observe a clear trend of decreasing MAE and MSE with increasing $\min(N_i^{(\mathrm{s})}, N_j^{(\mathrm{s})})$, with the most severe outliers for binary systems with very small $\min(N_i^{(\mathrm{s})}, N_j^{(\mathrm{s})})$.

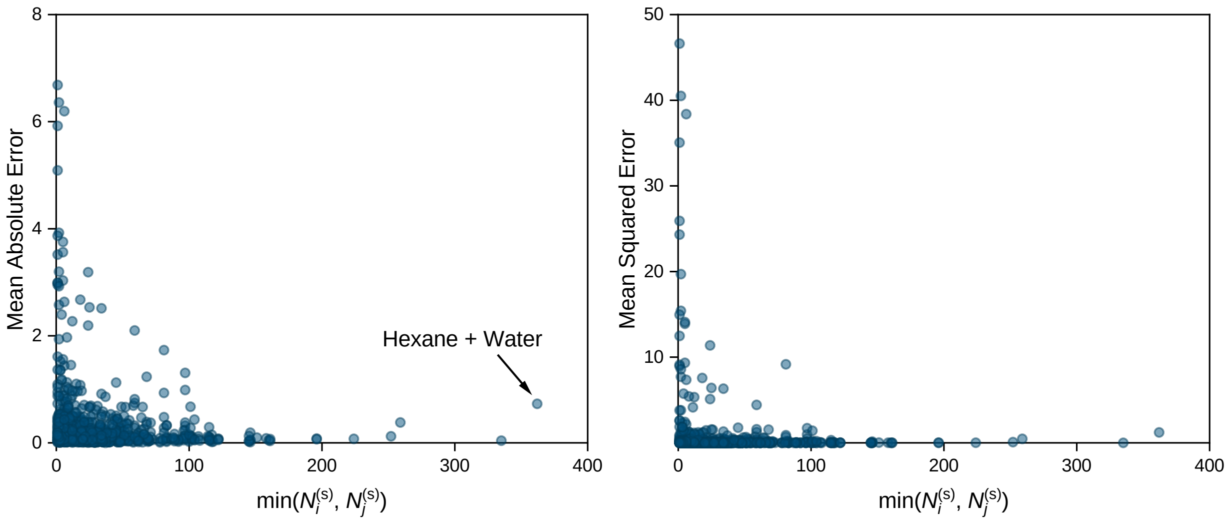

Figure S.6: Mean absolute error (MAE, left) and mean squared error (MSE, right) of the predictions of MCM-UNIQUAC for $\ln \gamma_{ij}$ in the binary systems from the test set as a function of $\min(N_i^{(s)}, N_j^{(s)})$, where $N_i^{(s)}$ ($N_j^{(s)}$) is the number of binary systems containing component i (j) in the training set. The deviations were calculated by comparing the predictions to the experimental data from the DDB. The shown ranges cover all systems from the test set.

Information on the 20 systems from the test set with the largest MAE is additionally summarized in Table S.1. Most of these systems include water, which is the most frequently studied component in our data set, and a second component that is very rarely studied and therefore rather poorly represented in the training set. We can therefore attribute the poor performance of MCM-UNIQUAC for some systems to two reasons: first, there are rather few training data points for at least one of the components, and second, water is one of the components (note that even for the system hexane + water, where for *both* components many training data were available, relatively large deviations are observed, cf. labeled point in Figure S.6). While the first reason is quite obvious for a model that relies only on the available mixture data, from which it infers, during the training, the characteristics (the features) of the constituent pure components, we can explain the second reason by the 'extreme' nature of water. Water can lead to both extremely small and extremely large activity coefficients, depending on the type of component it is mixed with. To fully capture this behavior, a complex and flexible model would be required, which could, however, easily be overfitting for less studied and less 'extreme' components. Our goal here was to develop a

comprehensive model with the greatest possible scope, which apparently comes at the cost of some deficiencies for aqueous systems. It will be interesting to develop more specified models in future work, e.g., concentrating on highly polar components, and compare the results to those of the wide-ranging model we provide here.

Table S.1: Binary systems $i-j$ from the test set with the largest mean absolute error (MAE) of the predictions with MCM-UNIQUAC. $N_i^{(s)}$ and $N_j^{(s)}$ denote the number of systems in the training set that include the component i or j, respectively.

Component i	Component j	$N_i^{(s)}$	$N_j^{(s)}$	MAE
2-Methylhexane	Water	1	382	6.69
Isoamyl propionate	Water	2	382	6.36
Bromocyclohexane	Water	6	382	6.20
4-Chloronitrobenzene	Water	1	382	5.92
m-Nitrotoluene	Water	1	382	5.09
2-Chloropropane	Water	2	382	3.92
1,4-Cyclohexadiene	Deuterium oxide	1	14	3.87
1-Phenyldodecane	Hexafluorobenzene	5	13	3.75
Piperidine	Water	5	382	3.56
Hexadecylcyclohexane	Phenol	1	81	3.52
Hydrogen fluoride	Water	2	382	3.20
Tetradecane	Water	24	382	3.19
Mono-n-butyl phosphate	Acetone	5	183	3.03
Hexadecylcyclohexane	Triethylene glycol	1	66	2.99
Hydrogen chloride	Water	1	382	2.99
Sulfur dioxide	Hexane	1	362	2.95
2-Methylheptane	N-Formylmorpholine	2	59	2.92
Di-n-propyl ether	Water	18	382	2.67
D-(+)-Limonene	Water	6	382	2.63
Xylene	Water	2	382	2.58

MCM-UNIQUAC based on Pair-Interaction Parameters ΔU

In Figure S.7, the results of a version of MCM-UNIQUAC based on the asymmetric pair-interaction parameters (ΔU), which are typically considered when UNIQUAC is applied, for the prediction of the $\ln \gamma_{ij}$ from the training and the test set are shown. For both sets, the MAE and the MSE averaged over binary systems are considered. The scores are slightly worse than that of the version of MCM-UNIQUAC discussed in the manuscript, which is

based on the symmetric pair-interaction energies U, cf. Figure 2 in the manuscript and Figure S.2.

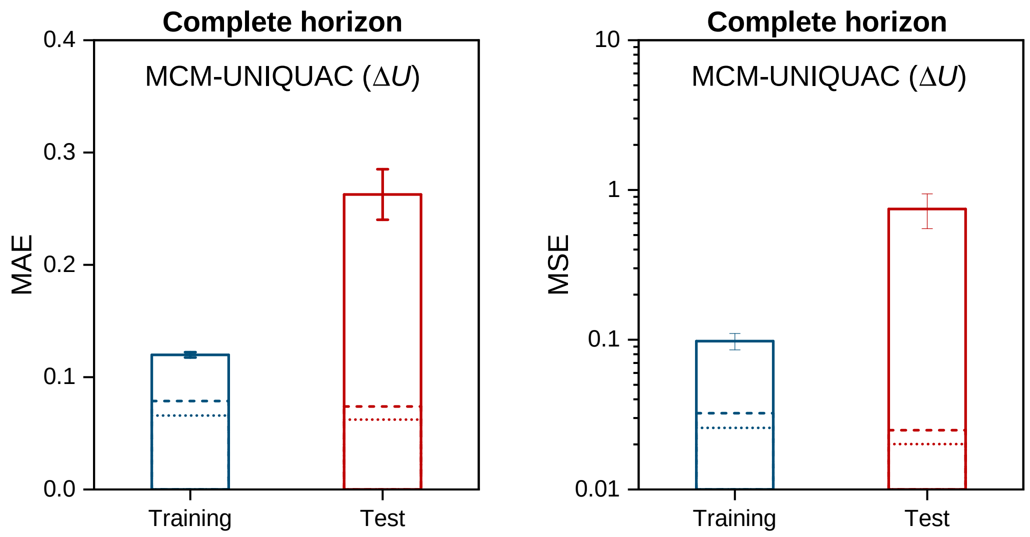

Figure S.7: Mean absolute error (MAE, left) and mean squared error (MSE, right) of a variant of MCM-UNIQUAC based on pair-interaction parameters (ΔU) on the training set and on the test set indicated as bars. The lines denote the baselines obtained by directly fitting UNIQUAC pair-interaction parameters (ΔU, dotted) or pair-interaction energies (U, dashed) to all available data points. Error bars denote standard errors of the means.

Complete UNIQUAC Parameter Set

In a separate file, we provide a *complete* set of the pair-interaction energies U (including uncertainties in the form of standard deviations) of UNIQUAC for all combinations of the considered 1146 components as predicted by MCM-UNIQUAC after training on *all* experimental data points, i.e., without withholding data points for validation and testing (no results for this scenario are shown above or in the manuscript). The hyperparameters were thereby set based on the model selection procedure to the values described above. From these pair-interaction energies, the commonly used pair-interaction parameters ΔU of UNIQUAC can easily be calculated following Eq. (3) in the manuscript. This parameter set can be used for the prediction of activity coefficients in any binary and multicomponent system of the considered components, even if no experimental data points on this system (and the

constituent binary subsystems) are available. However, we note that the predictions should be used with caution, in particular if components or systems are considered that have only been sparsely examined in experiments.

Studied Components

In the above mentioned file, we also provide an overview over all studied components, including CAS numbers (if available) and chemical formulae.

References

(1) Abrams, D. S.; Prausnitz, J. M. Statistical Thermodynamics of Liquid Mixtures: A New Expression for the Excess Gibbs Energy of Partly or Completely Miscible Systems. *AIChE Journal* **1975**, *21*, 116–128.

(2) Maurer, G.; Prausnitz, J. On the Derivation and Extension of the UNIQUAC Equation. *Fluid Phase Equilibria* **1978**, *2*, 91–99.

(3) Fredenslund, A.; Jones, R. L.; Prausnitz, J. M. Group-contribution Estimation of Activity Coefficients in Nonideal Liquid Mixtures. *AIChE Journal* **1975**, *21*, 1086–1099.

(4) Fredenslund, A.; Gmehling, J.; Rasmussen, P. *Vapor-Liquid Equilibria using UNIFAC, a Group-Contribution Method*; Elsevier: Amsterdam, The Netherlands, 1977.

(5) Onken, U.; Rarey-Nies, J.; Gmehling, J. The Dortmund Data Bank: A Computerized System for the Retrieval, Correlation, and Prediction of Thermodynamic Properties of Mixtures. *International Journal of Thermophysics* **1989**, 739–747.

(6) Jirasek, F.; Alves, R. A. S.; Damay, J.; Vandermeulen, R. A.; Bamler, R.; Bortz, M.; Mandt, S.; Kloft, M.; Hasse, H. Machine Learning in Thermodynamics: Prediction of

Activity Coefficients by Matrix Completion. *The Journal of Physical Chemistry Letters* **2020**, *11*, 981–985.

(7) Jirasek, F.; Burger, J.; Hasse, H. Application of NEAT for the Simulation of Liquid–Liquid Extraction Processes with Poorly Specified Feeds. *AIChE Journal* **2020**, e16826.

(8) Blei, D. M.; Kucukelbir, A.; McAuliffe, J. D. Variational inference: A Review for Statisticians. *Journal of the American Statistical Association* **2017**, *112*, 859–877.

(9) Zhang, C.; Bütepage, J.; Kjellström, H.; Mandt, S. Advances in Variational Inference. *IEEE Transactions on Pattern Analysis and Machine Intelligence* **2018**, *41*, 2008–2026.

(10) Kucukelbir, A.; Tran, D.; Ranganath, R.; Gelman, A.; Blei, D. M. Automatic Differentiation Variational Inference. *Journal of Machine Learning Research* **2017**, 1–45.

(11) Carpenter, B.; Gelman, A.; Hoffman, M. D.; Lee, D.; Goodrich, B.; Betancourt, M.; Brubaker, M.; Guo, J.; Li, P.; Riddell, A. Stan: A Probabilistic Programming Language. *Journal of Statistical Software* **2017**, 1–32.

(12) Weidlich, U.; Gmehling, J. A Modified UNIFAC Model. 1. Prediction of VLE, $\mathrm{h^E}$, and γ^∞. *Industrial & Engineering Chemistry Research* **1987**, *26*, 1372–1381.

(13) Constantinescu, D.; Gmehling, J. Further Development of Modified UNIFAC (Dortmund): Revision and Extension 6. *Journal of Chemical & Engineering Data* **2016**, *61*, 2738–2748.

(14) Hsieh, C.-M.; Lin, S.-T.; Vrabec, J. Considering the Dispersive Interactions in the COSMO-SAC Model for More Accurate Predictions of Fluid Phase Behavior. *Fluid Phase Equilibria* **2014**, *367*, 109–116.

(15) Bell, I. H.; Mickoleit, E.; Hsieh, C.-M.; Lin, S.-T.; Vrabec, J.; Breitkopf, C.; Jäger, A. A

Benchmark Open-Source Implementation of COSMO-SAC. *Journal of Chemical Theory and Computation* **2020**, *16*, 2635–2646.
